# Reduction in utilization of radiation for heterotopic ossification prophylaxis following acetabular surgery during the COVID-19 pandemic

**DOI:** 10.1007/s00590-026-04764-4

**Published:** 2026-05-22

**Authors:** Johnathan W. Riley, Isaac J. Spears III, Rowdy C. Lee, Julian C. Clark II, Cole Debevec, Josny Thimothee, Scott Weathersby, Priyanka V. Nehete, Peter N. Mittwede, Patrick F. Bergin

**Affiliations:** 1 Department of Orthopaedic Surgery and Rehabilitation, University of Mississippi Medical Center, Jackson, USA; 2 School of Medicine, University of Mississippi Medical Center, Jackson, USA; 3 Department of Orthopaedics, Louisiana State University Health Sciences Center Shreveport, Shreveport, USA; 4 Department of Emergency Medicine, Baylor Scott & White Medical Center - Temple, Temple, USA

**Keywords:** Heterotopic ossification, Prophylaxis, COVID-19, External beam radiation, Pandemic

## Abstract

**Purpose:**

Heterotopic ossification (HO) is common following acetabular surgery. Evidence comparing external beam radiation (XRT) and nonsteroidal anti-inflammatory drugs (NSAIDs) for HO prophylaxis remains conflicting. This study evaluated changes in XRT utilization during the COVID-19 pandemic and compared the efficacy of XRT versus NSAIDs.

**Methods:**

A retrospective review was conducted of 180 patients undergoing open acetabular fracture fixation at a level 1 trauma center from 2018 to 2021. High-risk patients received XRT or indomethacin if XRT was declined. Outcomes included prophylaxis method, severe (Brooker 3 or 4) HO, and HO resection.

**Results:**

XRT utilization decreased significantly during the pandemic (74.3% vs. 28.0%, *P* < 0.001). While severe HO rates remained similar (7.1% with XRT and 9.9% with NSAIDs) (*P* = 0.498), the only three HO resections were in the NSAIDs cohort.

**Conclusion:**

Despite reduced XRT use during COVID-19, HO outcomes were unchanged, suggesting both XRT and NSAIDs are effective prophylaxis options.

## Introduction

Heterotopic ossification (HO) is defined as the development of ectopic bone in soft tissues and can ultimately result in pain and functional impairment following operative fixation of acetabular fractures. The incidence of HO has been reported as low as 18% and as high as 90% among this population [1]. The iliofemoral and Kocher-Langenbeck approaches have been suggested to increase the risk for HO development as they involve an extensive dissection of the gluteal musculature [2, 3]. Additional reported risk factors include thoracic and abdominal trauma, male sex, T-type fractures, a high Injury Severity Score, delay in fracture fixation, and closed head injuries [2].

Two common methods for HO prophylaxis include external beam radiation therapy (XRT) and nonsteroidal anti-inflammatory drugs (NSAIDs), with indomethacin as the most common NSAID of choice. Although there is evidence suggesting that XRT and NSAIDs are both effective in preventing HO development [1, 3, 4], some studies have shown NSAIDs to have no significant effect on HO prevention compared to placebo [5–7]. Other literature has demonstrated that XRT is more effective at preventing severe HO than NSAIDs [8]. This distinction is significant, as the prevention of HO formation following fixation of acetabular fractures can reduce the rate of unplanned return to the operating room and decrease the risks and morbidities of this population.

The COVID-19 pandemic brought about unprecedented changes to the structure of healthcare and the personnel available to staff hospitals nationwide. Bayham et al. proposed the healthcare workforce decreased by 15% as a result of mandated school closure alone [9]. We suspect that restrictions placed on elective and nonessential procedures decreased the rate of XRT prophylaxis following operative fixation of acetabular fractures and was replaced with a course of indomethacin.

The purpose of this study was to evaluate if there was reduced utilization of XRT as prophylaxis for HO during the COVID-19 pandemic while seeking to compare the effectiveness of radiotherapy versus indomethacin in the prevention of severe HO and unplanned resections.

## Methods

A retrospective chart review analyzed patients who underwent open treatment for acetabular fracture under Current Procedural Terminology codes 27226, 27227 and 27228 at a level 1 trauma center over a 36 month period from January 2018 through January 2021. All patients were evaluated preoperatively with anteroposterior and Judet pelvic radiographs along with thin-cut computed tomographic scans of the pelvis with bone windows. Those with concomitant pelvic ring injuries were not excluded. Patients who underwent a posterior (Kocher-Langenbeck) approach were deemed high risk for HO development and subsequently referred for XRT postoperatively [3, 10]. Patients who declined XRT were prescribed a prophylactic course of indomethacin. Minimum radiographic follow-up was set at 12 weeks.

Patients who underwent XRT prophylaxis received a one-time dose of 700 centigray within 4 days of surgical fixation of their fracture. Conversely, there was not a standardized dosing regimen for patients electing to take indomethacin as prophylaxis. Dosages ranged from 75 to 150 mg daily for a duration of 14–90 days. However, a majority of patients were prescribed a course of 75 mg indomethacin orally for 42 days.

Demographic data including age, sex, height, weight, and body mass index (BMI, calculated by dividing patient weight in kilograms by the square of height in meters) were collected. Head injury at time of presentation, mechanism of injury, time to surgery, surgical approach, operative time, and operative blood loss were all noted. Follow-up measures consisted of HO prophylactic method, postoperative radiographs, unplanned reoperation, length of follow-up, and HO development. Postoperative radiographs were analyzed in a blinded fashion by a senior orthopaedic trainee and categorized based on the Brooker classification. Brooker class 1 and 2 were considered to be low-grade, while Brooker class 3 and 4 were considered to be high-grade [11].

March 20, 2020, was used to stratify patients into pre-pandemic and intra-pandemic groups. This date was selected due to its alignment with local quarantine restrictions as well as the implementation of social distancing measures that began to limit interactions among hospital staff and patients.

Primary outcomes included utilization of radiotherapy and indomethacin before and during the COVID-19 pandemic, development of high-grade HO before and during the COVID-19 pandemic, and rates of HO development among those treated with XRT or indomethacin prophylaxis. Secondary outcome measures included HO resection rates among those treated with XRT or indomethacin, along with the influence of various demographic and peri-operative variables on the development of HO including sex, BMI, presence of head injury, operative time, and operative blood loss. Statistical software used to analyze the data was SPSS Inc (Chicago, IL). The software was used to compare the results via chi-square tests, independent sample *t* tests, and analysis of variance where appropriate. Statistical significance was defined as a *P* value < 0.05. Ethical approval was obtained prior to performing research. No funding was received.

## Results

**Fig. 1:**
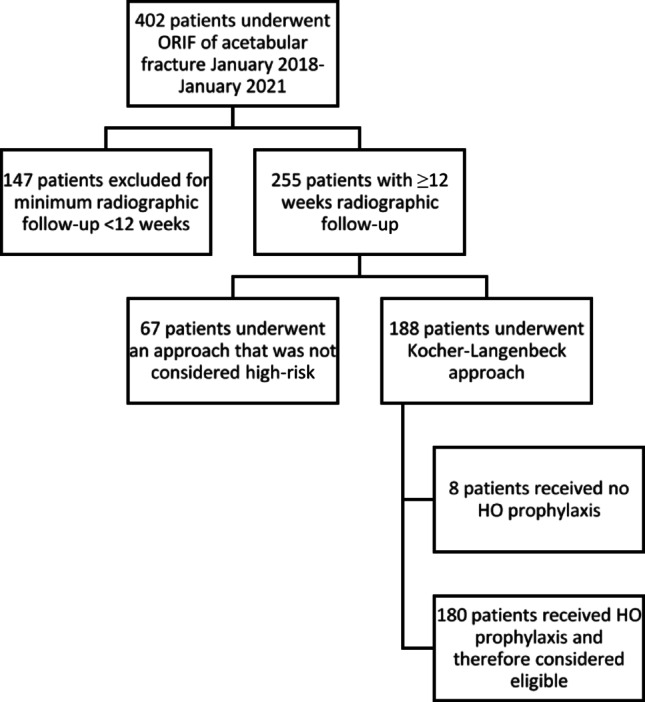
Flow chart depicting exclusions of original 402-patient cohort. ORIF—open reduction and internal fixation; HO—heterotopic ossification

During the 36 month period from January 2018 through January 2021, 402 patients were treated with open reduction and internal fixation for acetabular fractures. Of those, 147 patients were excluded for not having a minimum radiographic follow-up of 12 weeks, and 67 patients were excluded for receiving an approach other than a Kocher-Langenbeck. Of the remaining 188 patients, 8 were excluded for receiving no prophylaxis, which left 180 eligible patients (Fig. 1). The overall cohort had an average age of 37.7 years (range, 11–79, standard deviation +/- 15.2), an average BMI of 32.7 kg/m2 (range, 18.9–65.7, standard deviation +/- 8.9), and consisted of 107 (59.4%) males and 73 (40.6%) females. Head injury was present in 28 (15.6%) of patients. A majority of patients (90.0%) were involved in a motor vehicle or motorcycle accident.

**Fig. 2:**
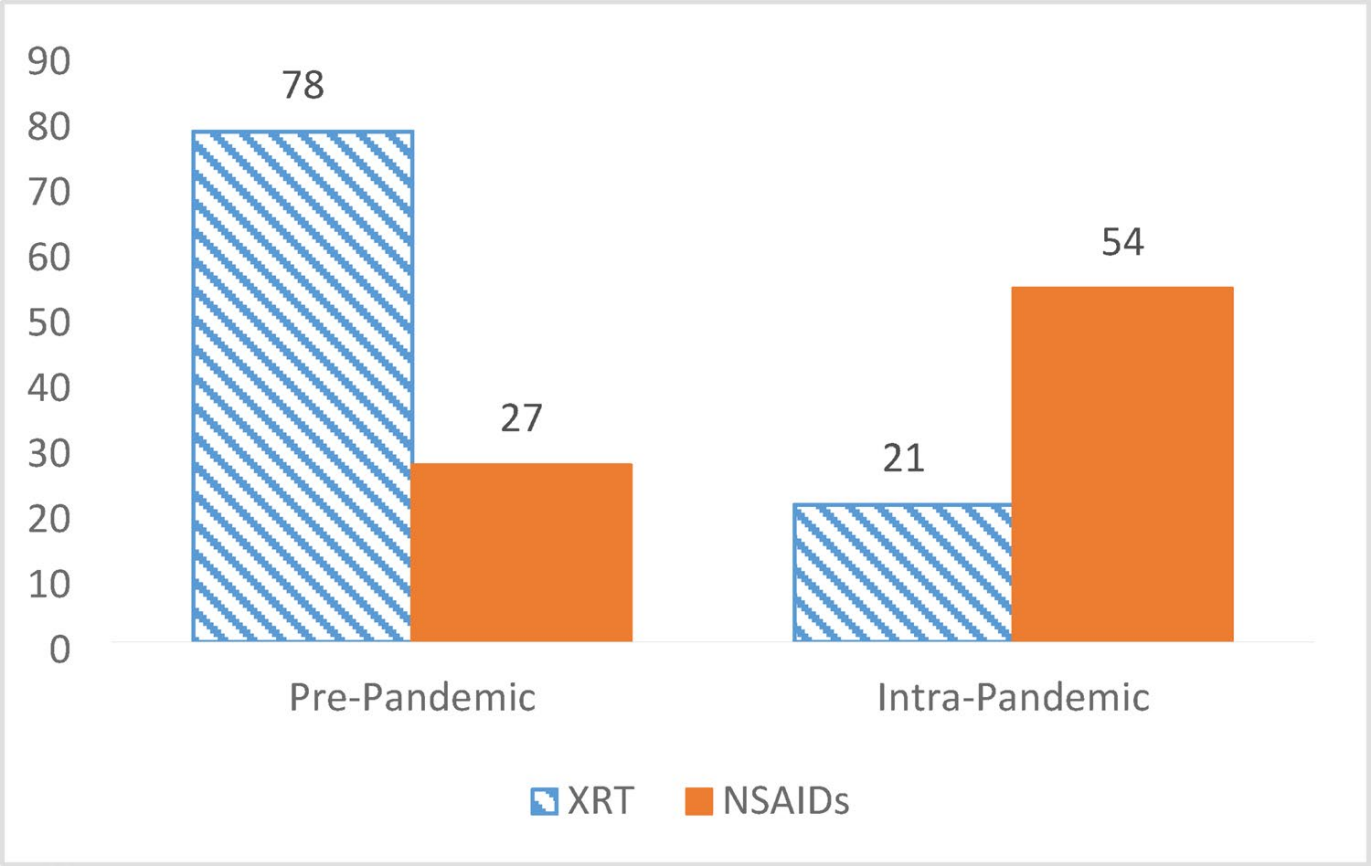
Bar chart showing decrease in XRT utilization and increase in NSAID use as prophylaxis for heterotopic ossification before and during the COVID-19 pandemic. XRT—external beam radiation; NSAID(s)—nonsteroidal anti-inflammatory drug(s)

105 patients were treated pre-pandemic, and 75 patients were treated intra-pandemic. XRT usage showed a significant decrease with 78 (74.3%) high-risk patients receiving XRT pre-pandemic and only 21 (28.0%) receiving XRT during the pandemic (*P* < 0.001). Conversely, indomethacin usage significantly increased, with 27 (25.7%) patients receiving NSAIDs pre-pandemic and 54 (72.0%) receiving NSAIDs intra-pandemic (*P* < 0.001) (Fig. 2). Pre-pandemic, 9 (8.6%) patients developed Brooker class 3 or 4 HO, while 6 (8.0%) developed similar disease process during the pandemic (*P* = 0.891). Overall, the XRT group had a 7.1% incidence of developing Brooker class 3 or 4 HO, while the indomethacin group had a 9.9% incidence (*P* = 0.498). Additionally, 3 (3.7%) patients in the indomethacin group underwent HO resection versus none in the XRT group (*P* = 0.053, RR = 1.038). Final radiographs were obtained at an average of 43.5 weeks postoperatively (range, 12–162, standard deviation +/- 33.2) and mean follow-up length was 43.5 weeks (range, 12-161.9, standard deviation +/- 33.2).

Patients averaged a 3-day wait from injury to surgery (range, 0–27, standard deviation +/- 3.2). Operative times averaged 266.2 min (range, 70–892, standard deviation +/- 134.0), and operative blood loss averaged 343.9 mL (range, 100–1300, standard deviation +/- 189.1) (Table 1). The presence of a head injury was found to have a statistically significant association with the development of severe HO (*P* = 0.047). Operative blood loss, operative time, BMI, sex, and mechanism of injury were not significantly related to development of high-grade HO. There was also no significant difference between those receiving XRT or indomethacin regarding patient age, sex, or presence of head injury.

**Table 1:** Demographic breakdown, time intervals, and operative details of pre-pandemic cohort and intra-pandemic cohort with *P*-value for significance of difference between cohorts

	Pre-pandemic patients	Intra-pandemic patients	*P*-value
Age in years at injury	38.8 (range, 14–79)	36.1 (range, 11–72)	0.239
Sex, Male (% cohort)	65 (61.9)	42 (56.0)	0.426
Sex, Female (% cohort)	40 (38.1)	33 (44.0)	
BMI	33.0 (range, 19.0-56.4)	32.4 (range, 18.9–65.7)	0.647
Days from injury to surgery	3.2 (range, 0–27)	2.9 (range, 0–10)	0.582
Operative time (minutes)	300.9 (range, 92–892)	217.5 (range, 70–526)	< 0.001
Estimated blood loss (mL)	354.3 (range, 100–1300)	329.3 (range, 100–1200)	0.384
Days to final radiograph	299.0 (range, 86-1105)	320.0 (range, 89-1135)	0.559

BMI Body mass index, mL Milliliters

## Discussion 

Development of heterotopic ossification following acetabular surgery has been closely associated with the Kocher-Langenbeck approach [3, 10]. Meta-analysis has shown the incidence of HO development following operative repair of acetabular fracture to be 25.6% [12]. Prophylaxis against HO development has been approached via two different strategies: radiation therapy [13, 14] and indomethacin [6, 15, 16]. Both methods have shown to be efficacious in the reduction of HO formation after acetabular fixation [2]; however, each has its own advantages and disadvantages. This institution specifically experienced a significant decrease in XRT for the prevention of HO with a consequent increase in indomethacin prophylaxis.

The COVID-19 pandemic presented a unique challenge regarding HO prophylaxis following acetabular surgery. This institution saw a decrease in XRT with 74.3% of high-risk patients receiving XRT pre-pandemic and only 28.0% receiving XRT during the pandemic (*P* < 0.001). Radiation therapy requires that a patient be transported from the ward to the radiation department along with the radiation personnel to perform the procedure [1]. During the pandemic, school closings were estimated to lead to the absence of 6–19% of relevant healthcare personnel [17]. While some healthcare labor occupations allow for remote work, absences due to COVID-19 likely affected the availability of personnel for postoperative XRT and contributed to its decreased utilization and replacement with indomethacin prophylaxis. Furthermore, there was an observed increase in health awareness among the general population during the COVID-19 pandemic, especially due to familial and social media influence [18]. An increase in health awareness could have steered patients away from receiving XRT when radiation oncology consultation discussed the risks of malignancy and infertility despite the dosage in pelvic radiation used for acetabular fracture being in a safe range regarding those adverse effects [19]. Even though some have suggested radiation therapy to be superior to indomethacin in the prevention of HO [1, 20], an increase in health awareness could have contributed to the decreased utilization in XRT seen at this institution.

XRT has been extensively studied as a method to reduce HO following acetabular surgery [1, 4, 13, 14, 20, 21] and was the main form of prophylaxis used at this institution prior to COVID-19. It is thought that external beam radiation disrupts mesenchymal cell differentiation and can reduce osteoblast production that is hypothesized to lead to HO formation [21]. Blokhuis and Frolke performed a systematic review showing a significantly lower percentage (*P* = 0.034) of patients treated with XRT developed HO compared to those treated with indomethacin and suggested that radiation therapy is a preferred method for the prevention of HO after operative treatment of acetabular fractures [1]. Kolbl et al. performed a randomized control trial showing that a 7 Gy (Gy) fraction is the most effective postoperative treatment schedule in prevention of clinically significant HO following hip replacement as compared to a single 5 Gy fraction or use of NSAIDs [20]. This study showed a difference that was not statistically significant in high-grade HO development before and after COVID-19 along with no statistically significant difference in high-grade HO development among those receiving XRT or indomethacin therapy. However, no patients among those receiving XRT required reoperation for excision of symptomatic heterotopic ossification.

Indomethacin has also been studied as a method to reduce HO following acetabular surgery [2, 4–7, 15, 16, 22]. Burd et al. performed a randomized trial comparing the effectiveness of radiation therapy and indomethacin to find that there was no significant difference in efficacy between the two prophylactic regimens [2]. Multiple other studies have shown no significant difference in indomethacin versus XRT prophylaxis [4, 23]. Other studies have shown no difference in HO development comparing indomethacin prophylaxis to placebo [5–7]. Indomethacin has also been associated with increased risk of long-bone nonunion [22] as well as radiographic nonunion of the posterior wall of the acetabulum [7]. This study showed no significant difference in XRT or indomethacin prophylaxis and the development of high-grade HO; however, the only patients that required unplanned reoperation for excision of symptomatic high-grade HO were in the indomethacin prophylaxis group.

One important consideration is patient adherence. If a patient agrees to receive XRT prophylaxis, a one-time dose of radiation is administered that is largely independent of patient-related factors. When a patient elects to forgo radiation therapy and chooses to take a course of indomethacin, patient adherence is crucial. Conversely, XRT is not without risks. XRT may increase patients’ lifetime risk of malignancy and may affect fertility in both sexes, though the radiation dose to the testicles or ipsilateral ovary averages lower than the dose required to permanently affect fertility. Men should be counseled on the risks of transient oligospermia and warned against fathering children within 9 weeks of XRT. Women should be advised on the risk of indeterminate damage to the ipsilateral ovary, though no defined contraception window is necessary in women [19].

Similar to the Mourad et al. retrospective analysis, we saw no significant correlation in high-grade HO development and operative blood loss, operative time, BMI, gender, or mechanism of injury [24]. However, the presence of head injury was significantly associated with severe HO development. The relationship between neurologic injury and HO has previously been reported and is well-accepted in existing literature [25].

This study is not without limitations. A large portion of patients did not have adequate follow-up, which reduced the number of eligible patients from which to draw comparisons between XRT and indomethacin prophylaxis for the prevention of HO following acetabular surgery. Long-term follow-up is an issue among the authors’ patient population, many of whom are from rural areas or are of lower socioeconomic status. Patient selection was also limited by the surgical approach in order to reduce confounding variables. Where possible, operative time was calculated only for the acetabular portion of the surgery in cases of polytrauma, however this was not possible in all cases and therefore some operative times include more than the acetabular fixation. Indomethacin prophylaxis dosages were not standardized within the group, and we also did not record patient compliance with taking this medication. Only Brooker classes 3 and 4 were assessed in this study, however Brooker classes 1 and 2 have the ability to eventually progress to Brooker class 3 or 4, which may have led to higher rates of severe HO if longer follow-up times were analyzed.

## Conclusion

While literature is decidedly varied as to the more effective prophylaxis for HO following acetabular surgery, radiotherapy and indomethacin should be considered as viable options and can be used on a case-by-case basis. The COVID-19 pandemic provided a unique environment at this institution in which the utilization of radiation therapy significantly decreased and indomethacin prophylaxis significantly increased without a significant change in rates of high-grade heterotopic ossification. Larger cohorts and randomized control trials should be performed on this topic to aid in the decision-making process for HO prophylaxis following acetabular surgery.

## Data Availability

De-identified research data is available upon request and completion of a Data Use Agreement.
